# The alkaline pectate lyase PEL168 of *Bacillus subtilis* heterologously expressed in *Pichia pastoris* is more stable and efficient for degumming ramie fiber

**DOI:** 10.1186/1472-6750-13-26

**Published:** 2013-03-19

**Authors:** Chengjie Zhang, Jia Yao, Cheng Zhou, Liangwei Mao, Guimin Zhang, Yanhe Ma

**Affiliations:** 1 College of Life Sciences, Hubei University, Wuhan 430062, China; 2 State Key Laboratory of Microbial Resources, Institute of Microbiology, Chinese Academy of Sciences, Beijing 100101, China

**Keywords:** *Bacillus subtilis*, Pectate lyase, *Pichia pastoris*, Glycosylation, Degumming, Codon optimization

## Abstract

**Background:**

The conventional degumming process of ramie with alkaline treatment at high temperature causes severe environmental pollution. Pectate lyases can be used to remove pectin from ramie in a degumming process with reduced environmental pollution and energy consumption. Pectate lyase PEL168 from *Bacillus subtilis* has been previously characterized and the protein structure was resolved. However, *Bacillus* is not a suitable host for pectate lyases during the degumming process since most *Bacillus* produce cellulases endogenously with a detrimental effect to the fiber. *Pichia pastoris*, which does not express endogenous cellulases and has high secretion capability, will be an ideal host for the expression. No previous work was reported concerning the heterologous expression of pectate lyase PEL168 in *P. pastoris* with an aim for industrial application in ramie bio-degumming.

**Results:**

The gene *pel168* was expressed in *P. pastoris* in this study. The recombinant protein PEL168 in *P. pastoris* (PEL168P) showed two bands of 48.6 kDa and 51.4 kDa on SDS-PAGE whereas the enzyme expressed in *E. coli* (PEL168E) was the same as predicted with a band of 46 kDa. Deglycosylation digestion suggested that PEL168P was glycosylated. The optimum reaction temperature of the two PEL168s was 50°C, and the optimum pH 9.5. After preincubation at 60°C for 20 min, PEL168E completely lost its activity, whereas PEL168P kept 26% of the residual activity. PEL168P had a specific activity of 1320 U/mg with a *K*_*m*_ of 0.09 mg/ml and a *V*_*max*_ of 18.13 μmol/min. K^+^, Li^+^, Ni^2+^ and Sr^2+^ showed little or no inhibitory effect on PEL168P activity, and Ca^2+^ enhanced enzyme activity by 38%. PEL168P can remove the pectin from ramie effectively in a degumming process. A 1.5 fold increase of PEL168 enzyme expression in *P. pastoris* was achieved by further codon optimization.

**Conclusions:**

Pectate lyase PEL168 with an available protein structure can be heterologously expressed in *P. pastoris*. The characterized recombinant PEL168P can be used to remove pectin from ramie efficiently and the expression level of PEL168 in *P. pastoris* was increased markedly by codon optimization. Therefore, PEL168 is an ideal candidate for further optimization and engineering for bio-degumming.

## Background

Ramie (*Boehmeria nivea*) produces one of the strongest and longest plant fibers that are used for clothing fabrics, industrial packaging, twines, cordages, canvas, car outfits, etc. However, despite excellent properties and diverse applications, ramie has failed to become a major textile crop mainly due to difficulties in processing the fiber. Natural ramie fibers contain gum-like material that has to be removed before most application. The conventional degumming process is performed with alkaline treatment at high temperature, which causes fiber damage while increasing energy consumption and severe environmental pollution.

Pectate lyases are a group of enzymes that catalyze hydrolysis of α-1, 4-glycosidic bond of pectin polymer. In industry, these enzymes, combined with other enzymes, can be used to efficiently remove ramie gum under mild conditions so that the use of harsh chemicals can be significantly reduced [1]. Hence, enzymatic degumming can serve as a good alternative to conventional degumming method, with a decreased environmental pollution and energy consumption as well as an improved yield and quality of degummed ramie [2]. For enzymatic degumming of fibers at an industrial scale, it is important to produce cost-efficient, effective and cellulase-free pectate lyase.

Pectate lyases have been isolated from various microbial sources such as yeasts, actinomycetes, bacteria and fungi. In application, alkaline pectate lyase is preferred for efficiently removing pectin from ramie, since pectin is more soluble in alkaline solution. Alkaline pectate lyases are produced predominantly by the genus *Bacillus *[3-5]. However, *Bacillus* is not a suitable host for pectate lyases during the degumming process since most *Bacillus* produce cellulases endogenously with a detrimental effect to the fiber. In contrast, *P. pastoris* has no endogenous cellulases expression and secrete heterologous proteins, serving as a good host for pectate lyase expression and application. In addition, proteins expressed by *P. pastoris* were sometimes N-glycosylated and had improved thermo-stability [6,7], which made *P. pastoris* more suitable for industrial application [8].

The genome of *Bacillus subtilis subsp. subtilis str*. 168 has been sequenced previously [9], and the pectate lyase gene *pel168* of this strain has been identified and expressed in *Escherichia coli* BL21 (DE3) for analysis of protein structure [10]. Five putative N-glycosylation sites on PEL168 can be deduced from the sequence. However, No previous work was reported concerning the heterologous expression in *P. pastoris* and the ramie degumming experiments by PEL168.

With a long-term goal to produce low-cost pectate lyase in industry application, structure based enzyme engineering is a major technical pathway to obtain optimized enzymes for industrial degumming. In the mean time, finding an ideal host for heterologous expression of the optimized enzyme is also essential for the large scale application. Therefore, we tested the expression of the alkaline pectate lyase gene *pel168* in *P. pastoris* and investigated the effectiveness of the recombinant enzyme PEL168 on degumming in this research. The data showed that the gene *pel168* can be expressed in *P. pastoris* and recombinant PEL168 can be used to remove pectin from ramie efficiently in degumming process. In order to improve the expression level, the *pel168* was further optimized according to the codon bias of *P. pastoris* to obtain 1.52 fold increase in extracellular activity. This is the first report that pectate lyase PEL168 can be used to remove pectin from ramie efficiently and that codon optimization was successfully used to increase the expression of pectate lyase in *P. pastoris*.

## Results

### Sequence analysis of pectate lyase PEL168

The nucleotide sequence of pectate lyase gene *pel168* from *B. subtilis* 168 was obtained from GenBank (accession number: AL009126). The estimated molecular mass of PEL168 was 46 kDa with a 21-residue signal peptide [11]. Five putative N-glycosylation sites (Asn-X-Thr/Ser) were found: three of them were near to the N terminus (N-48, N-127, N-184), and the other two were near the C terminus (N-345, N-351).

### Expression and deglycosylation of recombinant PEL168 expressed in *P. pastoris*

**Figure 1:**
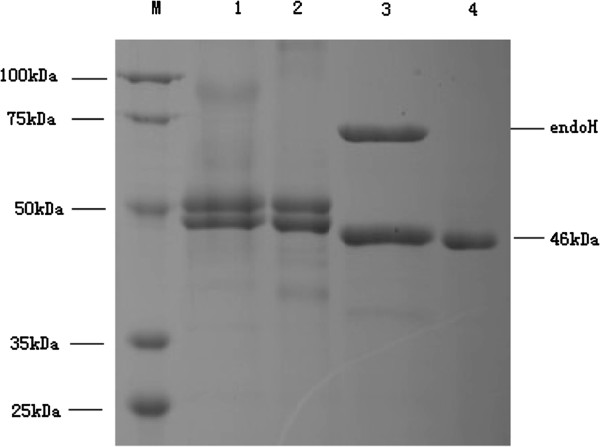
**SDS-PAGE of the pectate lyase protein.** Lane M, the protein molecular weight standards (100, 75, 50, 35 and 25 kDa); lane 1, concentrated PEL168P from supernatant; lane 2, purified PEL168P; lane 3, purified PEL168P treated by endoglycosidase H; lane 4, purified PEL168E.

The gene *pel168* of *B. subtilis* 168 was cloned into the *E. coli* expression vector pET28a and *P. pastoris* expression vector pHBM905A, respectively. To distinguish PEL168 expressed in *E. coli* and *P. pastoris*, the two enzymes were named PEL168E and PEL168P, respectively. The enzyme was purified and the purity was quantified by SDS-PAGE. Purified PEL168E showed one band with a molecular mass of 46 kDa (Figure 1), as predicted. However, PEL168P had two bands with molecular masses of 48.6 and 51.4 kDa, respectively (Figure 1), both larger than predicted. After a deglycosylation step by endoglycosidase H, only one band showed on SDS-PAGE, with the same size as the one expressed in *E. coli*, which suggested that the recombinant PEL168P had different degrees of glycosylation. Comparison of the molecular masses of the enzymes before and after deglycosylation showed that the percentages of glycosylation of the two fragments were 5% and 12%, respectively.

### Effect of cultivation temperature on PEL168P expression in *P. pastoris*

**Figure 2:**
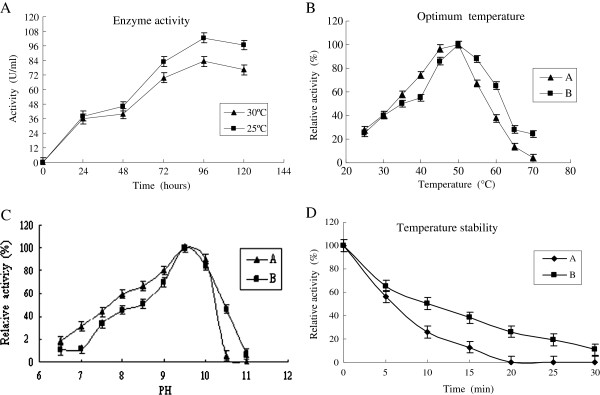
**A, Effect of culture age and cultivation temperature on pectate lyase activity in the culture medium of *P. pastoris*.** Pectate lyase activity at 25°C (▄ ). Pectate lyase activity at 30°C (▴ ). **B**, Effect of temperature on pectate lyase activity. Pectate lyase expressed in *P. pastoris* and *E. coli* was added to 50 mM Gly–NaOH buffer (pH 9.4) and incubated at different temperatures for 10 min. Maximum activity was set to 100%. A: recombinant PEL168E; and B: PEL168P. **C**, Effect of pH on pectate lyase activity. PEL168P and PEL168E were respectively incubated for 10 min at 50°C at the indicated pH values in 50 mM different buffer [Na_2_HPO_4_–citric acid (pH 4.0–6.0), sodium phosphate (pH 6.0–7.5), Tris–HCl (pH 7.5–8.8) and glycine–NaOH (8.8–10.6)]. Maximum activity was set to 100%. A, recombinant PEL168E; and B, PEL168P. **D**, Temperature stability of the purified pectate lyase. PEL168P and PEL168E were respectively incubated in the absence of substrate for 30 min at 60°C, and residual activity was measured as described in the methods section. The start-time enzyme activity was set to 100%. A: recombinant PEL168P; and B: PEL168E.

Transformant *P. pastoris* GS115-pel168 was cultured in BMMY liquid medium. The enzyme activity in culture supernatants was assayed daily for 5 days. As shown in Figure 2A, the time of optimal secretion of PEL168P was 4 days at two incubation temperatures. Maximum values of pectate lyase activity in culture supernatants were 102.56 and 83.12 U/ml at 25°C and 30°C (34.5 and 27.07 U/ml with A_235_ method), respectively. The culture kept at 25°C provided about 1.2-fold more activity than that kept at 30°C.

### Characterization of the pectate lyase PEL168

The activities of the recombinant PEL168s were measured at different temperatures (Figure 2B). The optimum reaction temperature of the two PEL168s (PEL168E and PEL168P) was both 50°C, and the two enzymes both showed more than 50% activity from 35 to 55°C. At 50°C, the optimum pH for the two PEL168s was 9.5, and both enzymes had more than 60% activity from pH 9 to 10 (Figure 2C). Therefore, PEL168 appears to be an alkaline-stable enzyme. The thermo-stability of these two pectate lyases was also investigated. The enzymes were pre-incubated without the substrate at 40, 50 and 60°C, respectively. For designated time periods, the two kinds of enzymes retained more than 85% of their initial activity at 40°C for 30 min. However, at 60°C, PEL168E completely lost its activity after 20 min pre-incubation, whereas PEL168P kept 26% of its initial activity with the same treatment (Figure 2D).

PEL168P had better thermo-stability and was more suitable for industrial application than PEL168E. Thus, we characterized PEL168P in detail*.* Under the optimum conditions as described above, the enzyme had a specific activity of 1320 U/mg (452 U/mg with A_235_ method) with a *K*_*m*_ of 0.09 mg/ml and a *V*_*max*_ of 18.13 μmol/min. Table 1 showed the effects of various metal ions and chemicals on the enzyme activity of PEL168P. K^+^, Li^+^, Ni^2+^ and Sr^2+^ showed little or no inhibition effect on the enzyme activity. Mn^2+^, Hg^2+^ and Cu^2+^ inhibited the most enzyme activity. Zn^2+^, Fe^3+^, Mg^2+^ and Co^2+^ reduced the enzyme activity by 35–55%. However, the enzyme activity was enhanced by 38% with Ca^2+^ addition. For the chemicals assayed, the purified enzyme completely lost its activity with SDS or EDTA. Tween-80, methanol, ethanol, isopropyl alcohol and glycerol showed slight or no inhibition effect on the enzyme activity, and DMSO showed 25% activation.

**Table 1:** Effects of metal ions and chemicals on the activity of the pectate lyase

**Metal ions or chemicals**	**Concentration**	**Relative activity(%)**
No addition	0 mM	100.0
K^**+**^ (KCl)	5 mM	102.8 ± 2.325
Zn^**2+**^ (ZnSO_4_)	5 mM	34.5 ± 3.657
Fe^**3+**^ (FeCl_3_)	5 mM	58.7 ± 0.562
Mg^**2+**^ (MgCl_2_)	5 mM	35.5 ± 1.246
Mn^**2+**^ (MnCl_2_)	5 mM	8.5 ± 1.21
Co^**2+**^ (CoCl_2_)	5 mM	47.0 ± 4.341
Hg^**2+**^ (HgCl_2_)	5 mM	0
Cu^**2+**^ (CuSO_4_)	5 mM	6.6 ± 1.564
Ca^**2+**^ (CaCl_2_)	5 mM	138.3 ± 3.564
Ni^**2+**^(NiSO_4_)	5 mM	81.8 ± 0.957
Li^**+**^ (LiCl)	5 mM	103.3 ± 2.451
Sr^**2+**^(SrCl_2_)	5 mM	104.1 ± 1.367
EDTA	5 mM	0
SDS	0.5%	0
Tween-80	0.5%	100.7 ± 3.124
DMSO	0.5%	124.8 ± 4.658
methanol	0.5%	98.3 ± 2.324
ethanol	0.5%	103.4 ± 1.246
isopropyl	0.5%	99.6 ± 2.154
alcohol	0.5%	101.5 ± 1.245

### Codon optimization of *pel168*

In order to further increase the expression levels of *pel*168 in *P. pastoris*, the sequence of *pel168* was optimized according to the codon bias of *P. pastoris*. Two hundred and ninety four nucleotides were substituted in the optimized sequence of *pel168s*. The G+C content of the original gene is 46.5%, as compared to 39.25% after codon optimization. The gene sequences before and after codon optimization were provided as Additional file 1. The expression analysis showed that transformants contained a single copy chromosomal integration of optimized *pel168* gene. And the transformant expressed 1.52 fold enzyme activity compared to the original *pel168*-containing strain under the same conditions, with enzyme activities in the supernatant of 166.74 U/ml and 109.7 U/ml (54.31 U/ml and 35.94 U/ml with A_235_ method), respectively.

### Effects of degumming by PEL168 expressed in *P. pastoris*

**Figure 3:**
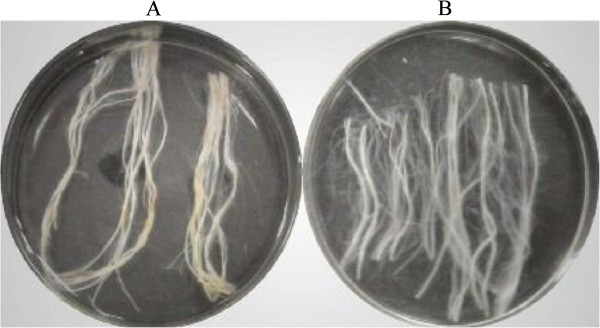
**Effect of degumming ramie fiber.** The ramie fibers were treated with PEL168P for 1 h. The controls had the same treatment without the addition of enzyme. A, control; and B, ramie fibers treated with PEL168P.

**Figure 4:**
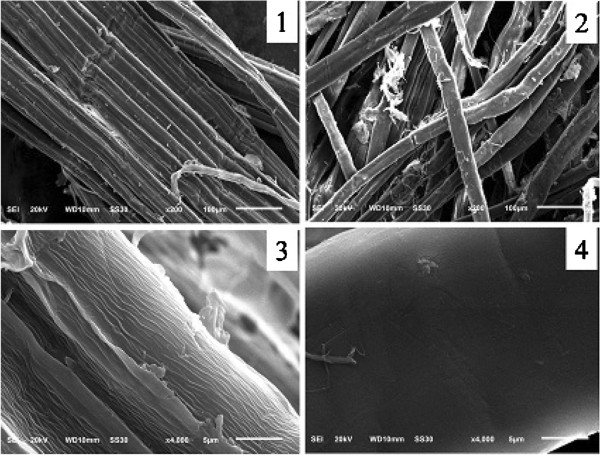
**Scanning electron microscope of degumming ramie fiber.** 1 (200×) and 3 (4000×), control; 2 (200×) and 4 (4000×), ramie fibers treated with PEL168P.

The degumming effects are shown in Figure 3. The ramie textile treated by PEL168P became softer, smoother and whiter, compared with that treated only with 0.05 M Gly–NaOH buffer. Dry weight measurement showed that enzyme-treated textile lost 15% weight. Residual content of gum was measured as 2% by alkaline treatment at high temperature. The textiles were also observed using a scanning electron microscope, and the enzyme-treated sample showed a smoother surface, suggesting that the gum-like material was mainly removed by pectate lyase PEL168P (Figure 4).

## Discussion

Gum-like materials from natural ramie fibers are soluble at high temperature and alkaline conditions, so chemical degumming is usually performed with alkaline treatment at high temperature, which causes serious environmental pollution. Enzymatic degumming serves as a good alternative to reduce pollution and cause less fiber damage. Therefore, developing a cost-efficient bio-degumming process of ramie by alkaline pectate lyase or bio-chemical degumming methods represents a major future trend of degumming industry. The low-cost of alkaline pectate lyase will be a key issue for accelerating bio-degumming application in the ramie treatment process. So it is important to get the cost-efficient and thermo-stable petate lyase with an improved expression level in an ideal host. *P. pastoris* is a preferred host for heterologous protein production with fast growing and facilitated gene manipulation. Moreover, it can also secrete heterologous protein efficiently. Thus, there is no need to break the cell and the enzyme secreted is easy to purify, which makes *P. pastoris* a more suitable host for industrial application [8]. Specifically, *P. pastoris* is an attractive host for pectate lyase expression because it does not produce endogenous cellulases that weaken the fiber of ramie. In this study, the pectate lyase gene *pel168* of *Bacillus subtilis* 168 was first successfully expressed in *P. pastoris* and the expression level was further increased by gene codon optimization.

The results in our study also indicated that the pectate lyase heterologously expressed in *P. pastoris* has different extents of glycosylation. It was reported that enzyme glycosylation in *P. pastoris* leads to better thermo-stability [6,7,12]. Although in the standard deglycosylation process, the pectate lyase must be denatured by boiling before being treated with endoglycosidase H, which makes it impossible to compare catalytic performance of the enzyme before and after deglycosylation. We described the influence of N-glycosylation on PEL168 by comparing the properties of the N-glycosylated with the non-glycosylated PEL168E from *E. coli.* The result showed that N-glycosylation did improve PEL168 thermo-stability. Thus, PEL168P was more stable in application and more suitable for degumming.

There are also some reports about the expression and characterization of alkaline pectate lyase. Yuan et al. cloned alkaline pectate lyase gene from *Xanthomonas campestris*[13] and *Streptomyces* sp. S27 [14], and expressed in *E. coli*, respectively. The optimum temperatures of the recombinant pectate lyases from *X. campestris* and *Streptomyces* sp. S27 are 30°C and 60°C, while the optimum pH are 9 and 10, respectively. A later effort by Solbak et al. leads to a candidate enzyme with an improved optimum temperature from 50°C to 70°C for the purpose of bioscouring [15]. Li et al. cloned an alkaline pectate gene from *Bacillus sp*. N16-5 and expressed it in *E. coli*. The optimum temperature and pH of the recombinant enzyme were 50°C and 11.5 [5]. Our initial trial to express alkaline pectate gene N16-5 in *P. pastoris* fails to get a high yield. We then turn our focus to the expression another alkaline pectate lyase which was originally isolated from *B. subtilis* 168 in *P. pastoris*. The recombinant PEL168P obtained in our current study displayed high efficiency at pH 9.5 and 50°C, with a specific activity (1320 U/mg) for removing the ramie pectin effectively in degumming process. This will provide a good starting point for further optimization of its thermo-stability and specific activity through protein engineering and strain engineering methods.

The codon optimization technique has been widely used to increase the expression levels of foreign proteins in *P. pastoris*[16,17], but not always successfully, and there is no related report about pectate lyases yet. So we tried the strategy described above in this study so as to increase the expression level. Expression and fermentation analysis showed optimized *pel168s* expressed 1.52 fold enzyme activity compared to the original *pel168*. This is the first report of codon optimization successfully increasing the expression of pectate lyase in *P. pastoris*.

The cultivation temperature is also an important factor for optimal production of heterologous proteins. Previous investigations have shown that lower cultivation temperature might improve the production of heterologous protein in *P. pastoris*, such as laccase [6] and xylanase [7]. This was also observed in our study. Culture at 25°C provided about 1.2-fold more activity than at 30°C. It has previously been shown that a lower cultivation temperature improves the production of heterologous protein in *E. coli*[18], which can be attributed to aggregation problems at higher temperatures.

In the degumming experiment, our result also showed that PEL168P can remove gum efficiently from ramie with a reduced process time. In the previous reports, the bio-degumming were usually performed by two ways. First, degumming was performed by isolated strains with high pectate lyase activity [1,2,19]. The degumming strains were inoculated on ramie fiber and incubated for a certain time, some degumming enzymes were produced with the growth of degumming strain so that the gum in the ramie fiber can be removed. For example, after 24 h of incubation with the isolated pectinolytic strain *B. pumilus* DKS1, the weight loss of the ramie fiber was found to be 25% under small scale [19]. This method requires longer time to perform degumming process because of the growth of degumming strains. Second, degumming was performed by enzymes from fermentation supernatant of isolated strains [20,21], which mainly includes pectate lyase and other polysaccharide-degrading enzymes. Culture supernatant of *Amycolata* sp. containing cellualse, xylanase and pectate lyase, reduced the gum content of ramie fibers by 30% within 15 h [20]. Brühlmann et al. [21] studied pectinolytic enzymes from actinomycetes for the degumming of ramie bast fibers. The gum content of the fibers decreased from 24% to 18% after 15 h treatment with crude enzyme preparations from a selection of actinomycetes at room temperature. This method also requires 15 h treatment since the activity of degumming enzyme isolated from wild strain is relatively low. From our data of SEM, the surface of the PEL168P-treated ramie looks smoother, which suggests that unique pectate lyase could also provide better degumming effects (Figure 4). As Compared to previous bio-degumming methods, the enzymatic degumming by PEL168P is more efficient and stable, with a minimal detrimental effect for fiber. Thus, PEL168P is a potential candidate to be used in bio-degumming process.

The protein structure information of pectate lyase PEL168 of *Bacillus subtilis* 168 is available now [10], which makes the enzyme rational design possible. Thus, PEL168P is a potential candidate for further improving the specific activity and reducing the cost so that it can be used in bio-degumming industry.

## Conclusions

In general, the alkaline pectate lyase gene *pel168* can be expressed in *P. pastoris* as a functional enzyme, and PEL168 can efficiently remove gum from ramie during the degumming process. In order to improve the expression level, the *pel168* was further optimized according to the codon bias of *P. pastoris* and resulted in 1.52 fold increase in extracellular activity. This is the first report that glycosylation of pectate lyase can improve thermo-stability and the expression of pectate lyase in *P. pastoris* is increased by codon optimization.

## Methods

### Strains

*B. subtilis subsp. subtilis str.* 168 was gifted by Prof. Li Yongquan from Zhejiang University, China. *E. coli* XL-Gold, BL21 (DE3) and *P. pastoris* GS115 were purchased from Invitrogen.

### Gene cloning and sequence analysis

The nucleotide sequence of pectate lyase gene *pel168* from *B. subtilis* 168 was obtained from GenBank (accession number: AL009126). Signal peptide was analyzed by Signal P 3.0 Server (http://www.cbs.dtu.dk/services/SignalP). N-glycosylation sites (Asn-X-Thr/Ser) were found by the NetNGlyC 1.0 Server (http://www.cbs.dtu.dk/services/NetNGlyc). The codon optimized gene (*pel168s*) was designed by DNAworks (http://helixweb.nih.gov/dnaworks/) and synthetized by Genscript, Nanjing, China. The sequence was also deposited in Genbank with accession number JQ655773.

### Construction of expression plasmid

The gene *pel168* of *B. subtilis* 168 was amplified by PCR. Primers P1, P2 and G1, G2 (Table 2) were designed for expression of *pel168* in *E. coli* and *P. pastoris*, respectively*.* The PCR products based on primers P_1_ and P_2_ were digested with *Bam*HI and *Not*I and then cloned into the *E. coli* expression vector pET28a, which was digested by the same restriction enzymes. The resultant plasmid was named pET28a-*pel168*. The PCR products based on primers G_1_ and G_2_ were digested with T_4_ DNA polymerase supplemented with dTTP for 20 min at 12°C to obtain *Not*I and *Cpo*I cohesive ends [22]. They ligated with *P. pastoris* expression vector pHBM905A [22], which was digested by *Not*I and *Cpo*I restriction enzymes. The resultant plasmid was named pHBM905A-*pel168*. The plasmid pHBM905A-*pel168s* was constructed by similar method based on primers H1 and H2 (Table 2).

**Table 2:** Primers used in this study

**Primers**	**Sequences**
**P1**	5^′^- CATTGGATCCATGAAAAAAGTGATGTTAGCTACGGC -3^′^
**P2**	5^′^- ATTCGCGGCCGCTTAATTTAATTTACCCGCACCCG -3^′^
**G1**	5^′^- GTCACAGCTGATTTAGGCCACCAGACGTTGG -3^′^
**G2**	5^′^- GGCCATTAATTTAATTTACCCGCACCCG -3^′^
**H1**	5^′^- GTCACTCGAGGCCGACTTGGGACA -3^′^
**H2**	5^′^- GGCCAGAATTCTTAGTTAAGTTTACCTGCTCCTGC-3^′^

### Transformation and gene expression

Plasmid pET28a-*pel168* was transformed into *E. coli* BL21 (DE3), and the transformant was cultured in 50 ml LB medium with kanamycin (50 μg/ml). IPTG (isopropylthiogalactoside) at a final concentration of 0.5 mM was added when OD_600_ reached 0.6. The culture was grown for 12 h at 18°C at 220 rpm. *P. pastoris* GS115 cells were prepared for transformation according to the manufacturer’s instructions (Invitrogen). Recombinant plasmid (10 μg) pHBM905A-*pel168* and pHBM905A-*pel168s* were linearized using *Sal*I restriction enzyme so that the ampicillin resistance gene and the ColE1 origin of replication were removed from both [22]. The linearized fragments were separately transformed into yeast cells by electroporation. Plasmid pHBM 905A was used to prepare a control strain. The transformants were inoculated on BMMY plate containing pectin and the methanol was added on the plate lid every 12 h. After 24 h culture and induction, transformants with the smallest halos that means single *pel168* gene integration in the genome were cultured in 50 ml BMGY liquid medium for 48 h. Cells were harvested by centrifugation and suspended in 25 ml BMMY liquid medium for gene expression. The cultures were incubated in BMMY for 96 h at 25°C at 220 rpm, and were supplemented with 1% methanol every 12 h. The pectate lyase activity was assayed every 24 h.

### Purification of recombinant pectate lyase

*E. coli* transformant cells were harvested by centrifugation at 1500 × *g* for 10 min, and washed twice with PBS. The cells were suspended in PBS and disrupted by ultrasonication. The crude pectate lyase supernatant was purified by Ni-NTA column and Superdex 75 column. The pectate lyase from *P. pastoris* was concentrated by an Amicon Ultra-15 centrifugal filter device (10 K MWCO; Millipore), and the protein sample was loaded on a Superdex 75 column. The molecular weight and purity of pectate lyase PEL168 were analyzed by SDS-PAGE (12%).

### Deglycosylation of PEL168 from *P. pastoris*

For enzymatic deglycosylation of N-linked glycans, 18 μl recombinant PEL168P was boiled for 10 min with 2 μl denaturing buffer and then incubated at 37°C for 1 h with 1 μl endoglycosidase H, according to the manufacturer’s instructions (New England Biolabs).

### Enzymatic characterization of pectate lyase

Pectate lyase activity was assayed with 0.25% (w/v) polygalacturonic acid (PGA; Sigma) as the substrate. Mixtures containing 20 μl diluted enzyme solution and 480 μl suspension of PGA in 0.05 M buffer were incubated at different temperatures for 10 min. The reducing sugar was determined by dinitrosalicylic acid procedure [23]. Galacturonic acid was used as a standard. One unit of enzyme activity was defined as the amount of enzyme capable of releasing 1 μmol of reducing sugar from PGA per minute under the assay conditions. Pectate lyase activity was also assayed by measuring the increase in absorbance at 235 nm (A_235_ method). One unit of enzyme activity was defined as the amount of enzyme capable of releasing 1 μmol unsaturated oligogalacturonic acid of from PGA per minute under the assay conditions.

The optimum pH of the pectate lyase was determined at 50°C using four different 0.05 M buffer systems: Na_2_HPO_4_–citric acid (pH 4.0–6.0), sodium phosphate (pH 6.0–7.5), Tris–HCl (pH 7.5–8.8) and glycine–NaOH (8.8–10.6). These experiments were performed in triplicate. The Statistical analyses of the experimental data were done with Microsoft Excel.

The kinetic parameters of PEL168P were determined at 50°C in 50 mM sodium glycine–NaOH buffer (pH 9.4), and reactions were conducted for 3 min with 0.1 μM PEL168P enzyme and PGA (0.05–0.25 mg/ml), in which the enzyme activity remained linear. The concentration of protein was measured by the method of Bradford with a protein assay kit from Bio-Rad, using bovine serum albumin as the standard.

To investigate the thermal stability of the enzyme, purified PEL168 was preincubated in the absence of substrates at 40, 50 and 60°C. Samples were taken at 5-min intervals during 30 min, and the residual pectate lyase activities were measured at 50°C for 10 min. The pH stability of PEL168P was also measured, and PEL168P was diluted in different buffers ranging from pH 4 to 10.5 and stored at 4°C for 12 h. The residual activities were measured at 50°C for 10 min.

The effects of various metal ions and chemicals on PEL168P activity were measured by adding different metal ions at final concentrations of 5 mM or chemicals at 0.5% (v/v) into the reaction system. The degree of inhibition or activation of enzyme activity was expressed as a percentage of enzyme activity in the control sample (no additional metal ion and chemical agent presented).

### Degumming by PEL168 expressed in *P. pastoris*

Degumming was performed according to Yang et al. [24]. The 5-g ramie fibers were boiled for 15 min in 250 ml water and transferred to 200 ml buffer of 0.05 M Gly–NaOH (pH 9.4) including 1 ml recombinant PEL168P, which is more than the requirement of the reaction. The mixture was incubated at 50°C for 1 h. Controls had the same treatment without the addition of PEL168P.

## Competing interests

All of authors declare no competing interests.

## Authors’ contributions

CJ Zhang carried out the pectate lyase gene expression in *P. pastoris* and helped with the writing of the manuscript; J Yao carried out the pectate lysase gene expression in *E.coli*; C Zhou participated in the sequcence alignment and glycosylation analysis; LW Mao carried out the protein purification. GM Zhang designed the whole study and drafted the manuscript. YHM participated in its design and coordination and helped to draft the manuscript. All authors read and approved the final manuscript.

## Supplementary Material

Additional file 1 **(1) The sequence of pectate lyase gene *pel168 *before codon optimization.** (2). The sequence of pectate lyase gene *pel168* after codon optimization. Click here for file
