# Supplementary material for: The alkaline pectate lyase PEL168 of Bacillus subtilis heterologously expressed in Pichia pastoris is more stable and efficient for degumming ramie fiber
Source: BMC Biotechnol. 2013 Mar 19;13:26. doi: 10.1186/1472-6750-13-26 (PMC3620942; doi:10.1186/1472-6750-13-26)
Supplement: Additional file 1 — (1) The sequence of pectate lyase gene pel168 before codon optimization. (2). The sequence of pectate lyase gene pel168 after codon optimization. [file 1472-6750-13-26-S1.doc]

**1, The sequence of pectate lyase gene *pel168* before codon optimization**

GCTGATTTAGGCCACCAGACGTTGGGATCCAATGATGGCTGGGGCGCGTACTCGACCGGCACGACAGGCGGATCAAAAGCATCCTCCTCAAATGTGTATACCGTCAGCAACAGAAACCAGCTTGTCTCGGCATTAGGGAAGGAAACGAACACAACGCCAAAAATCATTTATATCAAGGGAACGATTGACATGAACGTGGATGACAATCTGAAGCCGCTTGGCCTAAATGACTATAAAGATCCGGAGTATGATTTGGACAAATATTTGAAAGCCTATGATCCTAGCACATGGGGCAAAAAAGAGCCGTCGGGAACACAAGAAGAAGCGAGAGCACGCTCTCAGAAAAACCAAAAAGCACGGGTCATGGTGGATATCCCTGCAAACACGACGATCGTCGGTTCAGGGACTAACGCTAAAGTCGTGGGAGGAAACTTCCAAATCAAGAGTGATAACGTCATTATTCGCAACATTGAATTCCAGGATGCCTATGACTATTTTCCGCAATGGGATCCGACTGACGGAAGCTCAGGGAACTGGAACTCACAATACGACAACATCACGATAAACGGCGGCACACACATCTGGATTGATCACTGTACATTTAATGACGGTTCGCGTCCGGACAGCACATCACCGAAATATTATGGAAGAAAATATCAGCACCATGACGGCCAAACGGATGCTTCCAACGGTGCTAACTATATCACGATGTCCTACAACTATTATCACGATCATGATAAAAGCTCCATTTTCGGATCAAGTGACAGCAAAACCTCCGATGACGGCAAATTAAAAATTACGCTGCATCATAACCGCTATAAAAATATTGTCCAGCGCGCGCCGAGAGTCCGCTTCGGGCAAGTGCACGTATACAACAACTATTATGAAGGAAGCACAAGCTCTTCAAGTTATCCTTTTAGCTATGCATGGGGAATCGGAAAGTCATCTAAAATCTATGCCCAAAACAATGTCATTGACGTACCGGGACTGTCAGCTGCTAAAACGATCAGCGTATTCAGCGGGGGAACGGCTTTATATGACTCCGGCACGTTGCTGAACGGCACACAGATCAACGCATCGGCTGCAAACGGGCTGAGCTCTTCTGTCGGCTGGACGCCGTCTCTGCATGGATCGATTGATGCTTCTGCTAATGTGAAATCAAATGTTATAAATCAAGCGGGTGCGGGTAAATTAAATTAA

**2, The sequence of pectate lyase gene *pel168* after codon optimization**

GCCGACTTGGGACATCAGACACTTGGTTCTAATGACGGTTGGGGAGCCTATTCCACAGGTACTACAGGAGGTTCAAAAGCATCTTCATCTAACGTTTACACTGTTTCTAACAGAAATCAGTTGGTTTCTGCTTTGGGTAAAGAAACTAACACTACTCCAAAGATCATCTACATCAAGGGAACTATCGATATGAACGTTGATGACAATTTGAAGCCATTGGGTTTGAATGATTACAAAGACCCTGAATATGATTTGGACAAGTACTTGAAAGCTTATGATCCATCTACTTGGGGTAAAAAGGAGCCTTCTGGAACTCAAGAAGAGGCTAGAGCTAGATCCCAAAAGAATCAAAAGGCTAGAGTTATGGTTGATATCCCTGCTAACACTACTATTGTTGGTTCTGGAACTAATGCTAAGGTTGTTGGTGGAAACTTCCAAATTAAATCTGACAACGTTATCATCAGAAATATTGAGTTTCAAGATGCTTACGACTATTTCCCACAATGGGACCCAACTGACGGTTCTTCTGGAAACTGGAACTCTCAATACGATAACATCACTATTAATGGTGGAACTCATATTTGGATTGACCACTGTACTTTTAATGATGGTTCTAGACCAGACTCTACTTCTCCTAAGTACTATGGAAGAAAATACCAACATCACGATGGTCAAACTGACGCTTCTAACGGAGCTAACTACATCACTATGTCTTACAACTACTATCATGATCACGACAAGTCTTCTATTTTCGGTTCTTCTGATTCTAAAACTTCTGATGACGGAAAGTTGAAAATTACTTTGCATCACAACAGATACAAGAACATCGTTCAAAGAGCTCCAAGAGTTAGATTCGGTCAAGTTCATGTTTACAACAACTACTATGAAGGTTCTACTTCTTCTTCTTCTTACCCATTCTCTTATGCTTGGGGTATTGGAAAGTCTTCTAAAATCTACGCTCAAAACAACGTTATCGATGTTCCTGGTTTGTCTGCTGCTAAAACTATTTCTGTTTTCTCTGGTGGAACTGCTTTGTATGACTCTGGTACTTTGTTGAACGGAACTCAAATTAATGCTTCTGCTGCTAACGGTTTGTCTTCTTCTGTTGGATGGACTCCTTCTTTGCACGGTTCTATTGATGCATCCGCCAATGTCAAATCAAATGTCATCAACCAAGCAGGAGCAGGTAAACTTAACTAA
